# Outlining the Orientation Toward Socially Relevant Issues in Competitive R&D Funding Instruments

**DOI:** 10.3389/frma.2021.712839

**Published:** 2021-07-13

**Authors:** Andrea Orazio Spinello, Emanuela Reale, Antonio Zinilli

**Affiliations:** IRCrES - Research Institute on Sustainable Economic Growth, CNR - National Research Council of Italy, Rome, Italy

**Keywords:** project funding, policy instruments, evaluation, social relevance, competitive funding

## Abstract

While project-based funding in public R&D investments has grown in importance in all European countries over the last two decades, there is widespread concern among decision-makers about the actual orientation of project funding instruments to promote societal well-being. The capability of public R&D investment to improve the quality of citizens' lives implies the pursuit of “relevant” social objectives related to existing or emerging problems affecting individuals’ lives and society. Particularly, when referring to project-funded research, the question of “relevance” in research objectives recalls the never-ending debate over how to translate policymakers’ request for producing value from public investments in research activities into “usable results”. The manuscript explores, using recent data collected at European level on public R&D funding, the portfolio of research project funding policy instruments of various public research funding organizations (RFOs) in order to shed light on how and to what extent it is oriented to address socially relevant issues. The authors examine the characterization of the single project funding instruments, which are intended to incorporate the motivations and targeted goals of public action, and the RFOs that manage them. They specifically assume that the actual orientation of funding instruments, beyond the declared objectives, is influenced by some features related to their implementation operated by the RFOs, such as the importance given to specific evaluation criteria and the composition of the evaluation panels in the selection process of the funding beneficiaries.

## Introduction

One of the most important and recurring policy issues is the capability of public R&D investments to produce positive effects on science and society by addressing social problems and emerging challenges and providing solutions for improving the quality of citizens’ lives. Research priorities for European society’s needs, identified through foresight activities, shape the EU Framework Programmes (e.g., Horizon 2020), thus influencing funding allocation processes (Burgelman et al., 2014). Using the funding lever to steer the scholars’ research agendas toward addressing social issues through the development of targeted project funding instruments represents a viable policy option.

Between the two main types of public research funding schemes that the literature distinguishes – institutional funding and project-based funding (see Lepori et al., 2007; van Steen, 2012; Lepori, 2017) –, the latter is intended to be used for research oriented toward producing useful results rather than for curiosity-driven research. Resources distributed in a competitive way, addressing targeted research objectives, should improve the government’s ability to control the content of research activities developed by researchers, as well as the likelihood of R&D investment of producing effects on society (Braun, 2006). Since the 1980s, the push toward project funding allocations grew up, on the one hand to promote more selective methods of distribution in order to counter the stagnation of public spending on research, and on the other to promote the efficient use of public resources (Lepori et al., 2007). In response to new demands and opportunities, many countries have embarked on funding reforms, strengthening their strategic-planning capacity and paying closer attention to the social and economic environment, as well as the evolving patterns of relationships among stakeholders. This trend was reinforced in the 1990s. Government funding has been increased for mission-oriented and contract-based research, which is expected to be more reliant on output and performance criteria. Competitive mechanisms for allocation of public R&D funding have become more frequent in Research Funding Organizations (RFOs) portfolios, raising concerns about the equity of funding distribution based on performance (Hicks, 2012).

Having project funding instruments with objectives targeted toward topics of social relevance addresses the broader issue of relevance in science. On the one hand, defining a strategic orientation for research to societal challenges, with societal merit or impact included as a criterion for the decision of funding, responds to the society demand for expected value of research combined with a long-term view of socio-economic returns on research investments (Rip, 2003); on the other hand, setting priorities for scientific work is contested by scholars who believe that autonomy and freedom to choose the research questions to address are unavoidable characteristics of any scientific endeavor. Thus, tensions between the societal relevance of scientific work and research autonomy are at the core of several science policy studies (Demeritt, 2000; Scott, 2007; Gläser, 2016), affecting also the government capability to effectively steer the research system. Literature pointed out that the never-ending debate over how to combine the policy makers’ request of doing relevant research (producing usable results, not necessarily impact), and the need of scholars to maintain their autonomy and freedom of research, is still going on. The aforementioned preoccupation is also reflected in the proposal selection process; empirical evidence demonstrated the conservative nature of peer review, which frequently constrains the implementation of funding instruments oriented toward addressing topics of social relevance, because “relevance” generates resistance in the academic community (Scott, 2007). Thus, in project funding allocation, the capability of RFOs to properly manage the assessment of proposals to overcome the so-called relevance gap proved to be scarce; some authors provide suggestions in this regard (Nightingale and Scott, 2007), highlighting the importance of limiting academics’ monopoly on research funding panels and incorporating explicit relevance criteria into the peer review process, providing also reviewers with guidance on how to treat them.

Recent studies focused on how funding instruments could enhance the implementation of specific research approaches in order to foster societal transformations (Schneider et al., 2019) and emphasized the role of the funders in shaping scientific research toward a societal impact by “targeting” thematic orientation and foreseeing process of “enforcement” intended to ensure that researchers meet the targets (Aagaard et al., 2021). The purpose of this paper is not to delve into the efficiency or effectiveness of research systems in relation to promoting topics related to solving problems affecting citizens and society. Instead, the manuscript explores the portfolio of research project funding policy instruments of various European public RFOs, in order to shed light on what extent and how it is oriented toward socially relevant topics. To this aim, the authors use a new set of data that provides detailed information on several features characterizing the R&D governmental programmes mainly devoted to academic research. The authors analyze the characterization of the single project funding instruments, which are intended to incorporate the motivations and the targeted objectives of the public action. In this paper, they specifically assume that specific instrument features, related to their actual implementation – such as those related to the process for selecting beneficiaries, thus evaluation criteria and their importance, and the composition of evaluation panels – can have a role on characterizing the actual orientation of the single instruments.

## Materials and Methods

The analysis of the research funding orientation focuses on two main units of analysis: funding instruments and the RFOs that manage them.

Instruments are institutions that enable a policy to be operative, organizing the relationships between public power and targeted groups (Lascoumes and Les Gales, 2007). They demonstrate the actual characteristics of the policy design (Bleiklie, 2001) incorporating the motivations and the targeted objectives of the public action. They are the basic units of any governance mode (Capano et al., 2020), and widely used both in research policy studies and innovation studies to deepen the characteristics of policy mixes (Flanagan et al., 2011; Kern et al., 2019), and to understand the mission orientation of public funding.

RFOs are the agents which design and manage the R&D funding instruments. They retain control over the process of selection of beneficiaries of the R&D funding and are in charge of transferring resources for research activities. These entities have distinct missions, goals, and internal governance, implying varying levels of political influence and organizational autonomy (Lepori and Reale, 2019). One common trait is how to manage policymakers’ quest for relevant research, as well as the need to include “social relevance” in the objectives of the instruments and in the selection criteria, due to resistance from the scientific community in both basic and applied research (Braun, 2006). It was also noted that the differentiation of RFOs in the national context can lead to either a further broadening of objectives and strategies or a narrowing of goals and priorities (Whitley et al., 2018), which could lead to either greater flexibility of evaluation criteria or a stronger standardization of evaluation approaches. The latter outcome may be more likely if significant reductions in public R&D institutional funding are combined with an increasing reliance of the public science system on project funding allocations.

### Background Assumptions

Beyond the officially declared objectives, there is a gap between the policy formulation of the instrument, in which the original goal of the policymakers is described, and the implementation of the instrument, in which the “shaped goal” is put into action. (Reale and Seeber, 2013). The actual implementation of the instrument may direct its orientation toward unexpected directions.

Following Nightingale and Scott (2007), in this paper the authors assume that – beyond the declared orientation – the actual capability of project funding instruments to address issues of social relevance is primarily related to how the aims and objectives are put into actions by the managing RFOs through the decisions on the criteria used in the selection processes and on the composition of the evaluation panel in charge of selecting the funding beneficiaries.

As a result, they establish the two empirical prepositions on which their approach is based:

a. the more heterogeneous and flexible are the criteria driving the ex-ante assessment of the proposals, the greater is the possibility to use the funding instrument to address purposes other than those established by their formal objectives, because the importance of the criteria in the selection process is neither high nor low.
b. the more the composition of the evaluation panel includes mix of academics and external non-academic expert, the more panelists can have room in the implementation of the selection criteria on topics of social relevance of funding instruments.

Both of the aforementioned effects are influenced by tensions in the relationships between RFOs managing project funding and the government; RFOs struggle to maintain their space of maneuver and possibility to pursue their own objectives independent the government steering. As a result, key elements for understanding project funding portfolios beyond the formal objectives must also refer to the instruments’ implementation features.

### Data Sources

Investigating project funding instruments is a difficult task due to a lack of systematic and robust data that can support strong assumptions. Attempts have been made to follow the implementation of project funding by RFOs, as well as the types and modes of allocation (Lepori et al., 2007; Potì and Reale, 2007), but data constraints did not allow for a deeper understanding of the instruments’ portfolio characteristics using measurable items. The authors attempt to fill this gap in this paper by utilizing data derived from a large-scale study on public research funding supported by the EU Commission – PREF, refined and deepened with official documents extracted from the EFIL database, one of the H2020-RISIS2 project’s facilities.

PREF is a dataset derived from a large-scale study on public research funding supported by the Joint Research Center of the European Commission. By combining quantitative data and descriptors concerning allocation modes and criteria, as well as information on the flow of public funding and the RFOs managing funding, the project created a systematic methodological framework for analyzing public research funding systems in UE, associated, and accession countries (Lepori, 2017; Reale, 2017). Data collection followed the GBARD data collection rules (OECD, 2002, chapter 8) and covered the entire period from 2000 to 2014. Methodologically, the capability to measure project funding allocation proved to be reliable enough to allow further investigation (Lepori et al., 2018); furthermore, analyses performed on the dataset show that, aside from differences between European countries and between agencies within countries, project funding increased over time, both in absolute value and as a percentage of total government funding (Reale, 2017). Thus, data are suitable to be used for addressing the question whether the implementation of government R&D funding through project-based allocation is appropriate for addressing social problems and innovation.

For the purpose of the paper, the authors chose 146 competitive R&D funding instruments from 11 Western European countries – Austria, Denmark, France, Germany, Italy, Norway, Portugal, Sweden, Switzerland, The Netherlands and United Kingdom (Table 1), and considered data from the most recent available years, 2013–2014, since the data are completer and more reliable. The sample reflects the diversity of Western European countries and addresses the need for complete and robust data for the exploratory tests.

**TABLE 1:** Total instruments included in the analysis by country.

Country	AT	CH	DE	DK	FR	IT	NL	NO	PT	SE	UK	Total
Instruments	8	7	11	11	14	8	3	11	5	6	62	146

EFIL – European dataset of public R&D funding instruments is one of the new datasets included in the H2020-RISIS2 project. Its goal is to provide users with the ability to investigate public R&D funding in Europe at the level of funding instruments and RFOs. The database is still being built, and the data on funding instruments includes a parallel collection of official textual documents related to the instruments (calls, applicant guidelines, evaluation reports), in order to allow for the development of text analyses. The use of text analyses is expected to result in the detection of keywords in order to delve deeper into aspects of policy development and R&D funding orientation.

### Descriptors

A set of descriptors has been extracted to characterize the different ways in which social relevance can be implemented. In this regard, it is necessary to refer to the policy objective of the instruments as well as other elements such as: the presence of agencies with the specific mission of sustaining social relevant research, the presence of social relevance in the evaluation criteria of knowledge-oriented instruments, and the preference for expert-dominated selection panels.

Thus, the descriptors used in this study refer to:

i. RFO classification based on definitions derived from the literature in the field (Lepori, 2017), which allow them to be described in terms of autonomy and specialization.
The dataset distinguishes among RFOs that are functionally part of the public administration (research/science ministry; sectoral ministries); those that have a large degree of independence in managing their activities (innovation agencies; research councils; sectoral RFOs; higher education agencies); public research organizations whose primary mission is to perform R&D activities, but also can carry out some funding agency activities.
ii. Descriptors of funding instruments in each RFO (formal orientation of the project funding instrument; composition of the decision-making bodies entitled to carry out the selection process, allocation criteria for the projects evaluation).

Concerning the formal orientation of project funding instruments toward specific research objectives, the authors distinguished three broad groups of funding instruments (Lepori, 2017): (i) “economic innovation instruments,” i.e., instruments oriented toward pre-competitive development and the creation of market value, that cover the domain 06 of NABS classification^1^ (Industrial production and technology) and can be related to research on Key Enabling Technologies (KETs); (ii) instruments devoted to the general advancement of knowledge, i.e., instruments financing the curiosity-driven research, which broadly correspond to schemes without an explicit topic in the NABS classification (NABS12); (iii) “policy instruments,” i.e., instruments focused on research on existing or emerging problems in society which cover multiple domains, broadly corresponding to the NABS categories 01-05 (Exploration and exploitation of the Earth, Environment, Exploration and exploitation of space, Transport, telecommunication and other infrastructures, Energy), 07-11 (Health, Agriculture, Education, Culture, recreation, religion and mass media, Political and social systems, structures and processes) and 14 (Defense)^2^. This last category should include research on socially relevant topics, such as the one involving the Societal Grand Challenges (SGCs).

The descriptor on the composition of the decision-making bodies entitled to implement the selection process distinguished either composed by academics, experts, or with mixed composition.

Finally, each funding instrument has been characterized in terms of relative importance of the criteria considered by the panels for the projects’ assessment. The instruments received three different scores from national experts recruited by the PREF project, which scaled the importance of the evaluation criteria used for project funding assignment, specifically “academic quality”, “topicality to instruments subject”, and “potential for economic innovation and public/private cooperation.” Each criterion’s importance has been rated using the following scale: 5 = very important; 4 = important; 3 = moderately important; 2 = of little importance; 1 = unimportant. As a result of the assignment of the scores, cases of both the presence of one or two overriding criteria and the equal relevance of all three have emerged. It’s worth noting that the authors considered the method used to decompose the instruments in the PREF dataset, which is based on granularity to the point that scores are often assigned to aggregations of a small number of sub-schemes with similar characteristics but probably slightly different evaluation criteria. To overcome the latter constraint, the authors chose homogeneous project funding instruments relevant to national research activities, excluding instruments labeled as “transnational research” because they cover schemes with different orientations and evaluation criteria.

### Official Documentation

Exploration of the orientation of the research funding instruments based either in part or mostly on descriptors derived primarily from expert opinion may yield skewed results. In fact, this method reflects the broader issue of the experts’ production of scores, which are likely disputable measures for which different experts may disagree on single scoring assignments (Aksnes et al., 2017).

For this reason, the authors developed an original method to determine whether the global level of orientation in terms of the importance of assessment criteria would have been consistent with the features revealed by a text analysis of 46 selected calls for proposals from five countries chosen for the study – France (12 calls out of 14 instruments), Italy (4 out of 8), Sweden (6 out of 6), Switzerland (7 out of 7), United Kingdom (17 out of 62) – extracted from the official documentation archive linked to the EFIL database. Countries were chosen based on their size, the presence of various types of RFOs, funding instruments with varying degrees of orientation, and the quality of the data collected. The most recent available calls for proposals have been analyzed for each national instrument, particularly the sections describing the general overview of the instrument, the objectives and the criteria and rules of assessment. In case of non-availability of the calls, webpages containing the instrument’s description and information on how the proposals are assessed have been used. The majority of the documents were in English, with only a small percentage of them having been translated from the original language to English in order to perform the text analysis.

Taking into account the instruments’ granularity in the dataset used, only the “generic” calls related to the schemes, containing the general guidelines for project assessment (usually matching with distinctive evaluation rules by the funding agency), were taken into account, and specific calls were used only in the rare cases where a generic one was not available, i.e., for a few French instruments.

### Procedures

The study used an exploratory approach, articulated in three stages, to describe the orientation of project funding instruments in the selected countries.

More specifically, the authors performed: i) descriptive analyses on the single characteristics of the instruments (formal orientation of the instrument by RFO classification; composition of the decision-making body by formal orientation of the instrument; importance of assessment criteria by orientation of the instrument); ii) Multiple Correspondence Analysis (MCA) to investigate the pattern of relationships between the categorical variables used to classify the instruments and types of RFOs. Indeed, MCA allows extrapolating patterns across a set of variables described by single components. These components are considered latent unobserved variables that indicate the maximum variance of a set of other variables.

Finally, in order to control the reliability of MCA, in which experts’ scores were also used, the authors used official documentation from the archive associated to the EFIL database, to perform a text analysis, which yielded the most frequently occurring words in the instruments’ calls for proposals, indicating the prevalence of one or more relevant elements concerning the instruments’ objectives and the rules and criteria for evaluating proposals.

## Results

### Descriptive Statistics

**FIGURE 1:**
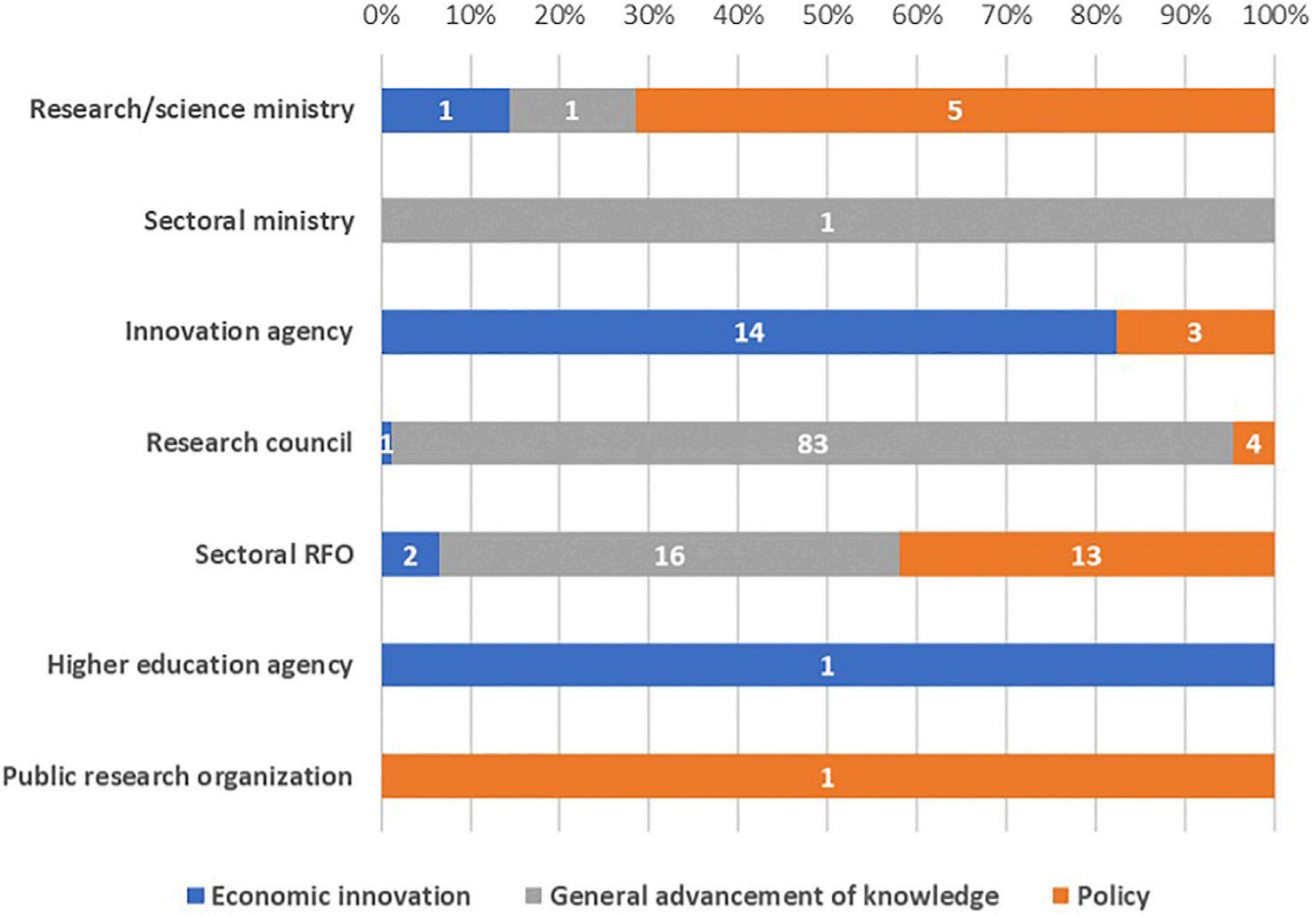
Formal orientation of funding instrument by RFO classification (a.v. of single funding instruments).

Funding instruments are classified into three broad categories: (i) instruments aimed at economic innovation and the creation of market value; (ii) instruments devoted to the general advancement of knowledge; and (iii) policy-oriented instruments that cover multiple domains and are intended to address topics that are closely related to existing or emerging problems in society. Only 26 (12%) of the 146 instruments examined are policy oriented. The vast majority (69%) are formally devoted to the general advancement of knowledge, thus mainly curiosity-driven research. Figure 1 illustrates the thematic orientation of project funding instruments implemented by various types of national RFOs in various countries, as outlined in formal documents. Data show that different types of RFO have varying degrees of “specialization” in terms of the instruments they manage: Innovation Agencies with economic innovation-oriented instruments, Research Councils with non-oriented instruments, Sectoral RFOs with a mix of policy-oriented and non-oriented instruments. Half of the total policy-oriented instruments is managed by Sectoral RFOs.

**FIGURE 2:**
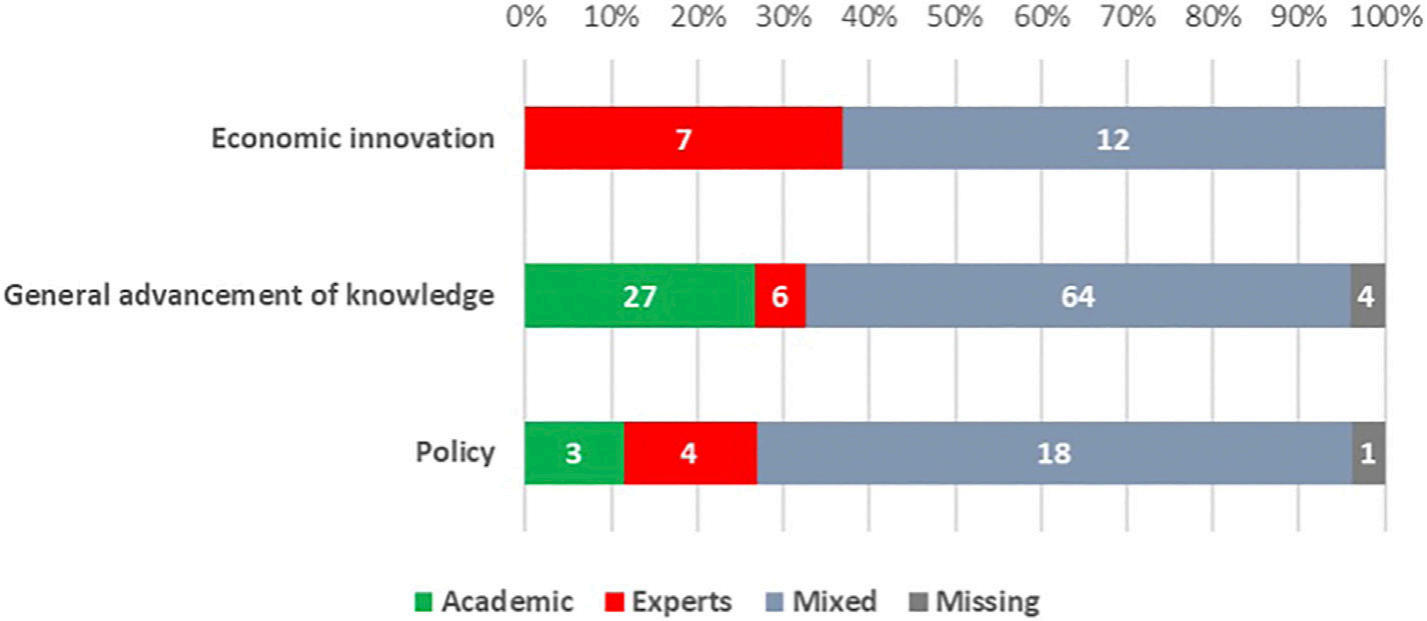
Type of decision-making body by formal orientation of the instrument. (a.v. of single funding instruments).

Figure 2 describes the composition of the decision-making bodies in charge of assessing the project proposals submitted by thematic orientation of the funding instrument. “Academic” means a panel completely or largely composed by academics; “Experts” means a panel mainly composed by persons outside the academia with a professional expertise on the topics of the call; “Mixed” signals a mixed composition of the panel balancing academics and experts. It is clear that the mixed composition predominates for all thematic orientations of the funding instruments (69% of policy-oriented and 63% of non-oriented and economic innovation-oriented instruments), while panels involving only experts are present in few cases, above all for instruments oriented to economic innovation.

The following two tables show the importance of allocation criteria in terms of the funding instruments’ orientation. Table 2 shows the percentage of instruments by thematic orientation for which the importance of the assessment criteria (academic quality; topicality; economic innovation) was rated higher than three (“important” or “very important”) by the PREF project experts. Table 3 shows the percentage of instruments in which importance of the assessment criteria was rated less than three (“of little importance” or “unimportant”). While instruments devoted to economic innovation and non-oriented ones, on the one hand, emphasize the importance of assessment criteria relating to “economic innovation” and “academic quality,” respectively; on the other hand, instruments oriented toward policy issues show the more heterogeneous situation when it comes to higher values, as the percentage of instruments scoring high criteria referring to academic quality is close to the percentage of the instruments scoring high criteria for topicality. Furthermore, when compared to other instruments, policy-oriented instruments have fewer low evaluation scores for topicality, and 23% of them have low assessment criteria for economic innovation.

**TABLE 2:** Importance of assessment criteria (score >3) by formal orientation of the instrument. Note: NL instruments and 1 DE instrument have some missing data on assessment criteria.

Thematic orientation	Academic quality *score > 3*	Topicality *score > 3*	Economic innovation *score > 3*
Economic innovation	31.6%	52.6%	94.7%
General advancement of knowledge	98.0%	63.4%	43.6%
Policy	73.1%	80.8%	57.7%

**TABLE 3:** Importance of assessment criteria (score <3) by formal orientation of the instrument. Note: NL instruments and 1 DE instrument have some missing data on assessment criteria.

Thematic orientation	Academic quality *score < 3*	Topicality *score < 3*	Economic innovation *score < 3*
Economic innovation	21.1%	10.5%	0.0%
General advancement of knowledge	1.0%	20.8%	21.8%
Policy	7.7%	3.8%	23.1%

### Multiple Correspondence Analysis

The MCA was carried out in order to determine what the most appropriate components are for characterizing project funding instrument portfolios in terms of orientation, taking into account all of the descriptors examined in the preceding paragraph.

**FIGURE 3:**
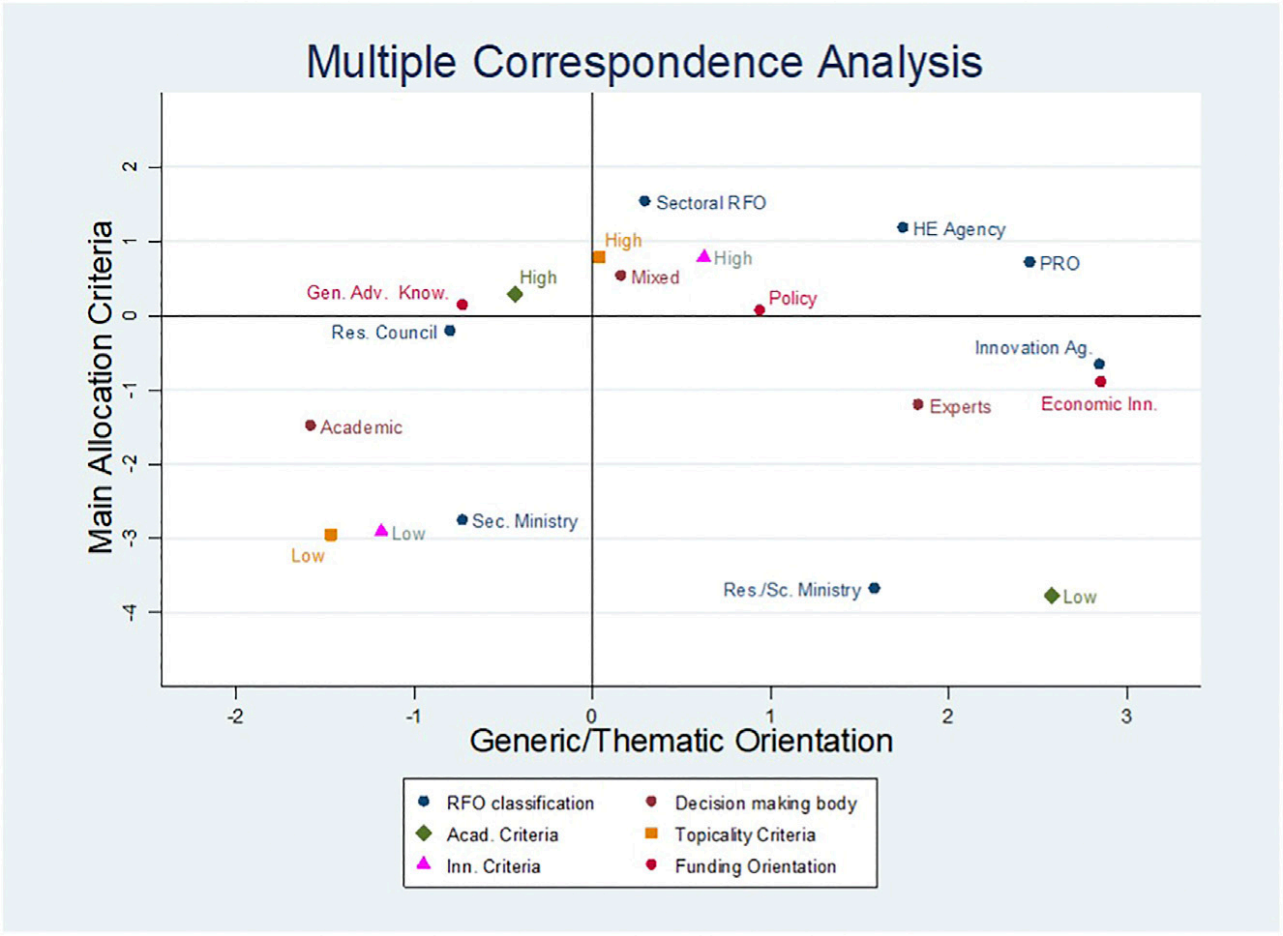
MCA coordinate plot.

MCA has a standard configuration, with the main axis (factor 1) explaining the majority of the data and thus representing the most significant component (Figure 3). Factors are bipolar, which means that each one has two opposing groups of modalities that define each factor and allow it to be characterised.

In the first factor (47 percent of inertia), that can be named “Generic vs. Thematic Orientation,” there is a presence of instruments that are not oriented toward specific goals on one side (managed by research councils, first and foremost, and characterized by panels whose composition is dominated by academics); on the other side, instruments with thematic orientation that characterized the other types of agencies, with the innovation agency and panels composed of non-academic experts having special relevance. Policy orientation is positioned on the horizontal line, indicating that it is little discriminating. It is clear that policy orientation and mixed decision making body have no significant.

The second factor (20 percent of inertia), that can be named “Main Allocation Criteria”, refers to the importance of the allocation criteria used for instrument evaluation, distinguishing instruments with high ratings on one side from instruments with low ratings on the other. Low values rating the importance of “innovation” and “topicality” are associated with the National Sector Ministry, whereas low levels of academic quality are associated with oriented instruments. High academic quality criteria scores are close to instruments with a generic orientation; policy orientation and mixed panel composition have no significant contribution to the two dimensions.

In summary, the first dimension explains the importance of oriented instruments vs. non-oriented ones, with research councils being the most important type of RFO associated with the latter. The second component allows to understand that non-oriented research is associated with low levels of importance for topicality and innovation criteria, whereas innovation-oriented instruments are associated with low levels of importance for academic criteria. Since policy instruments are not associated with low or high ratings of importance of any criteria, thus different combinations might produce an implementation of R&D funding allocation far from the general objective that the instruments are supposed to address.

### Text Analysis

Through a text analysis, a procedure used for extracting meaningful information from corpuses of text, 46 selected calls for proposals from the sample of 146 instruments were analyzed to check the reliability of the MCA. Aside from controlling for MCA robustness, word cloud models are extremely useful for analyzing policy instruments with various orientations and managed by various agencies, revealing the instruments “shaped” objectives.

**FIGURE 4:**
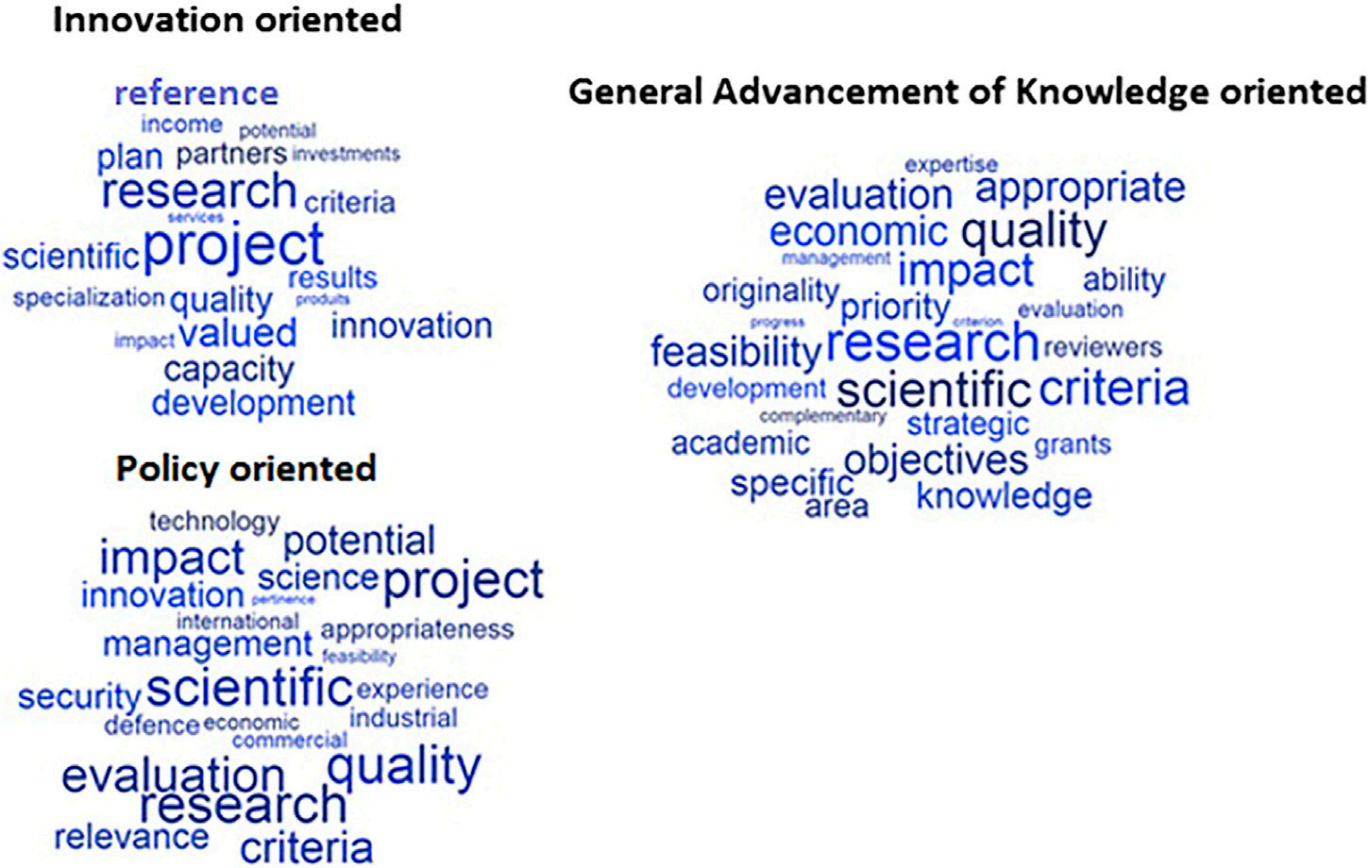
Word Clouds of funding instruments by formal orientation of the instrument.

The word corpus was standardised to allow comparison between different types of instrument orientation, and only words above the 95th percentile, were considered for the study. The authors used word clouds to identify the most frequent words within the selected calls, which were grouped by formal orientation; the larger the word size, the more frequent the word is in the document (Figure 4).

The most frequently used words in the calls for proposals of instruments with an innovation orientation are “research”, “project”, “reference,” “innovation,” “capacity,” and ”development.” “Research,” “scientific,” “quality,” and “impact” are among the most frequently used words in instruments devoted to the general advancement of knowledge. Finally, for policy-oriented instruments, the most frequently used words are “quality,” “scientific,” “research,” “evaluation,” and “impact”, all of which appear in large font size, indicating a higher frequency. In both of the latter two cases, the word heterogeneity is greater than in the former, and it is possible to notice a high recurrence of words like “quality,” “impact” and “evaluation.”

The findings do not contradict the evidence of MCA: while the differences between instruments are very clear when we look at the words related to the formal orientation of the instruments, the differences are not as clear when we look at the wording used for the evaluation criteria, with the exception of innovation-oriented schemes.

**FIGURE 5:**
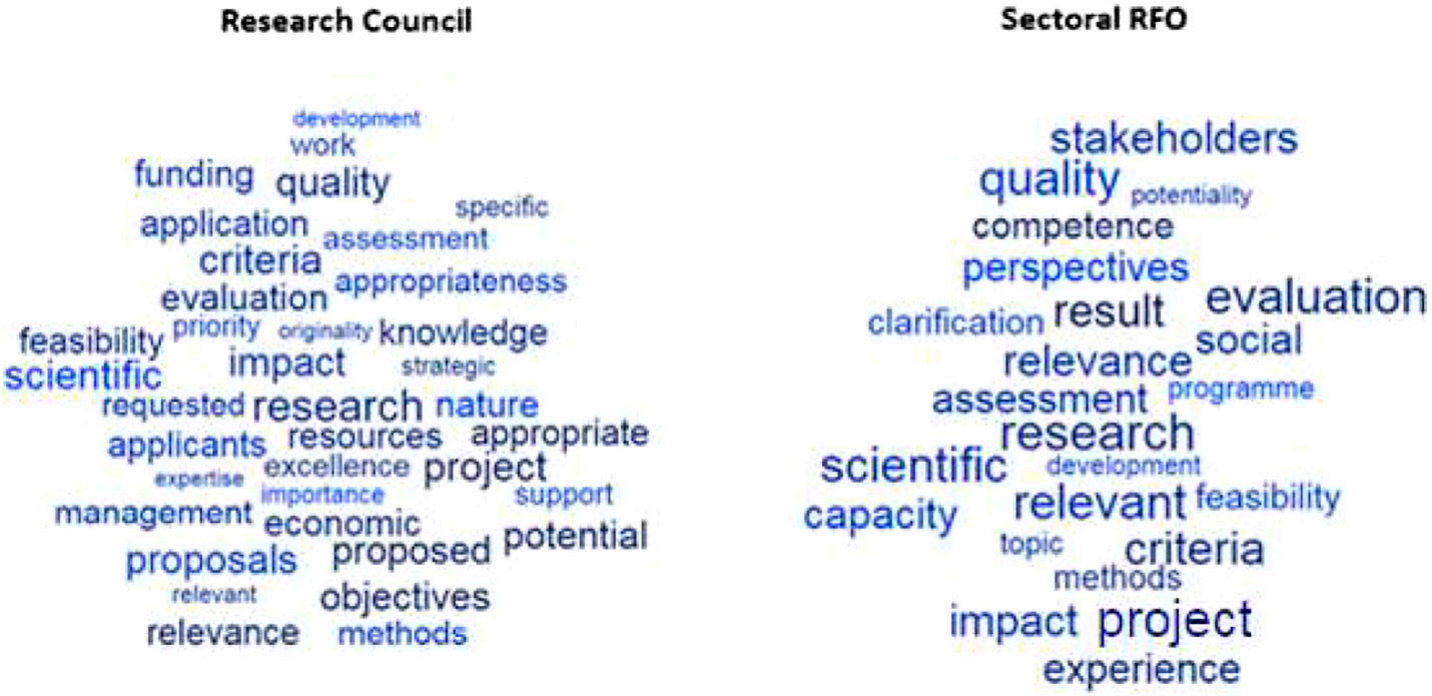
Word Clouds of funding instruments with no orientation and with a policy orientation by Research Council and Sectoral RFO.

Figure 5 examines instruments’ calls for proposals with no orientation and with policy orientation managed by research councils and sectoral RFOs. The other RFOs were not tested because they manage a very small number of instruments with the mentioned orientations.

The analysis confirms a high degree of wording heterogeneity, with significant overlap of recurrent words between the instruments managed by the two types of RFOs. However, sectoral RFOs have some recurring words with a higher importance (frequency) than research councils; moreover, there are words of instruments managed by sectoral RFOs that do not emerge in instruments managed by research councils (“stakeholders”, “perspectives”) or that are significantly more important in the former than in the latter (“impact”, “quality”, “relevance”, “experience”).

Taken together, MCA results and the text analysis suggest the reliability of the experts’ assessments with their scoring assignments. Text analysis also served to highlight the heterogeneity of the wording used in the drafting of the research instruments’ calls for proposals, which allows for an effective room for maneuver for the RFOs on policy implementation. The textual analysis contributed to a better understanding of the flexibility related to the actual orientation of the instruments and resulted in additional specifications, adding value to the MCA results. Further investigations could be carried out with the help of ontology analyses.

## Discussion

Since the 2000s, project-based funding in public R&D investment has grown in all European countries (Lepori et al., 2007; Reale, 2017). The rationale behind this trend – which is occurring at different rate and pace across all the European countries – was that improving competitive allocation mechanisms would allow for better research performance and more efficient use of funding resources by selecting the best research groups, promoting some subjects or research themes, supporting structural changes in knowledge production modes, and improving cooperation and competition among research groups (Geuna, 2001; Braun, 2006). Scholars have made several attempts over the years to control assumptions about the positive (Aghion et al., 2010; Wang et al., 2018) and negative effects of competitive funding on the performance of R&D systems (Laudel, 2006; Heinze, 2008; Reale and Zinilli, 2017). Other lines of investigation revealed the presence of tensions between competitive funding and research practices of scholars’ communities (Gläser and Laudel, 2016), which are further exacerbated by the reduction of the level of public resources allocated for R&D, resulting in unintended consequences such as the use of competitive funding for different tasks and the contamination of institutional funding with objectives designed for project funding (Gläser, 2016; Franssen et al., 2018) or to favor senior researchers and males at the expense of young researchers and females (Wang et al., 2018).

Aside from the issue of R&D system competition, project funding allocation can be investigated in the context of the relevance and value of public investment in research policy, especially at a time when policymakers are concerned about the low prominence of research activities oriented toward socially relevant objectives to answer societal questions. In this regard, EU policy recently emphasizes the so-called mission-oriented approach to improving the directionality of public R&I policies in order to promote innovation and well-being (Mazzuccato, 2018).

This paper addressed the problem of the orientation of government R&D project funding instruments toward topics of social relevance that are supposed to contribute to the societal well-being. The manuscript proposed an exploratory methodology that goes beyond simply analyzing policy instrument orientation as it emerges from formal objectives, and it also considers the characteristics of the instruments that emerge during their actual implementation, particularly in the beneficiary selection process, resulting in the modulation of the importance of the evaluation criteria and the composition of the evaluation panel.

The investigation revealed that funding instruments aimed at socially relevant issues are not widespread in the European countries under analysis. The importance of project funding orientation toward general advancement of knowledge, i.e., curiosity-driven research, remains strong. Based on the evidence from this study, it is not excluded in principle that instruments with some generic orientation or no orientation can address research activities that include topics of social relevance, with the effect depending on the design of the evaluation criteria and the panel composition.

As to the RFOs portfolio, we observed that Research Councils have primarily instruments with no thematic orientation toward socially relevant issues, but the evaluation criteria are flexible enough to allow for an implementation that may even score high the capability of the proposals to address objectives dealing with relevant policy problems. On the contrary, non-academic selection criteria are very important in funding instruments managed by sectoral RFOs, but the calls for proposals are characterized by a high degree of wording heterogeneity. In this regard, innovation agencies are those whose implementation is generally consistent with the formal orientation of the funding instruments. A preliminary explanation could be that RFOs with general missions – research councils first, implement project funding instruments based on “their core business” (Braun, 2006), attempting to maintain their independence from government steering. Instead, agencies with a specific mission, such as the innovation agencies, are characterized by instruments in which research is expected to address problems that are specifically devoted to creating added value and impact on the economy and society, and evaluation procedures are more focused on selecting projects that are oriented toward those objectives. One significant implication is that differences in project funding instruments between countries are related to the establishment of specialized agencies for managing instruments addressing socially relevant topics or to entrusting research councils with the task of managing such schemes.

Summing up, the evidence from the tests performed does not contradict the paper's empirical prepositions (see par. 2.1). The literature shows that research funding arrangements have been largely analyzed in terms of consequences on beneficiaries and to a lesser extent as to the consequences they produce on research practices. In this paper the authors instead focus on a relatively unexplored issue concerning decision-makers' design and implementation of project funding instruments. This different perspective has a specific value contributing to single out problems that may arise at the decision-making level, such as phenomena related to legal traditions and path dependency that may affect the way in which policy goals are shaped, as well as authority relationships between actors involved in the selection process.

### Limitations, Future Research, and Implications

Mapping the characteristics of the RFO portfolios with indicators addressing the importance given to the evaluation criteria, as well as the panel composition, can show the level to which project funding actual implementation can improve competition within the R&D system addressing socially relevant issues. The findings are preliminary attempts to understand how public policies are implemented across European countries using descriptors related to the design and implementation of project funding instruments; additional work is required to refine the descriptors and build indicators on both funding instruments and the position of RFOs as actors implementing R&D policies.

The MCA results suggest that policy-oriented instruments require more in-depth analysis to deepen implementation using evaluation criteria as evidence, as well as additional checks whether there are any biases affecting the scoring of evaluation criteria produced by experts and used in the analysis.

The method developed for this study may help in shedding light on the characteristics of national R&D funding systems, as well as the roles that project funding plays in European RFO portfolios. This perspective has the specific value of recognizing correlated problems that may arise at the decision-making level, which may affect how policy goals are shaped and achieved.

Further studies could be enriched by bibliometric analyses on the results and outputs of funding instruments, which could provide an additional element to address the research topic.

Finally, some implications of our findings can be outlined as to their consequences for different levels of analysis of project funding instruments. As to the *micro* level, analyses based solely on formal objectives of the programs are incapable of revealing their actual orientation toward socially relevant issues. On the contrary, combining indicators on different features (e.g., policy objectives, evaluation criteria in knowledge-oriented instruments, mission of the agencies and experts involved in the evaluation panels) is important for depicting them and comparing instruments across countries.

As to the *meso* level of analysis, the findings of this investigation confirm the importance of RFOs as key decision-makers for project funding implementation; additionally, data provide further evidence that there are various ways in which RFOs delineate their “space of political and scientific interests” in research funding, balancing the demand for relevance of research and the improvement of control from policymakers (Braun, 2006). One mean is to retain the political control over the availability of funding resources and the freedom of choice in the selection processes.

At *macro* policy level, the policy implementation of the funding instrument may differ from the original political goal because of technical gap in the design of the policy instruments and political gap in the presentation of the desired objectives. Instruments are flexible means that are shaped by policymakers’ goals and priorities, but when implemented, they are “far from being fully controlled by policy makers” (Reale and Seeber, 2013, 142). Therefore, understanding the extent to which R&D governmental programmes are actually oriented toward socially relevant goals, needs a comprehensive analysis, including also how the instruments are put into actions through the selection of the beneficiaries.

## Data Availability

The data supporting the conclusions of this article are available from the corresponding author on request. EFIL data will be publicly available for research purposes soon.

## Footnotes

1 The classification for analyzing public funding of research and development (R&D) on the basis of socio-economic objectives.

2 Although also research aimed at technological innovation may also be aimed at solving societal problems, the descriptor distinguishes between the instruments primarily devoted to the pre-competitive development, the curiosity-driven research and the remainder of oriented research. In this regard, the reference to the NABS classification is made explicit to ensure that there are no thematic overlaps.
